# Estimation of the Percentage of US Patients With Cancer Who Are Eligible for Immune Checkpoint Inhibitor Drugs

**DOI:** 10.1001/jamanetworkopen.2020.0423

**Published:** 2020-03-09

**Authors:** Alyson Haslam, Jennifer Gill, Vinay Prasad

**Affiliations:** 1 Division of Hematology and Oncology, Knight Cancer Institute, Oregon Health and Science University, Portland; 2 Now with Center for Health Sciences, Oklahoma State University, Tulsa; 3 Department of Public Health and Preventive Medicine, Oregon Health & Science University, Portland; 4 Division of General Medicine, Department of Medicine, Oregon Health & Science University, Portland

## Abstract

This cross-sectional study estimates the percentage of US patients with cancer who are eligible for immune checkpoint inhibitor drugs.

## Introduction

Immune checkpoint inhibitor (ICI) drugs have gained popularity in oncology because of their ability to boost a person’s immune response against cancer cells.^1^ We recently estimated that 43.6% of US patients with cancer are eligible for ICI therapy, and up to 12.5% of patients respond to it.^2^ However, those were best-case estimates, and postmarketing studies for several of these drugs have failed to show improvement in overall survival or progression-free survival.^3^ The US Food and Drug Administration (FDA) has revised some ICI drug labels, and future changes may follow.^4^ Accordingly, we sought to reestimate the eligibility of ICIs, taking into consideration recent FDA label changes and results of postmarketing studies.

## Methods

In this cross-sectional study, we used prior estimates of eligibility and response,^2^ based on American Cancer Society’s Cancer Facts and Figures and FDA drug labels (2011 through August 2018). We updated these estimates to reflect drug approvals through June 30, 2019. Indications for drugs previously included in the data set that have since failed to meet postmarketing obligations were removed from the total estimates.

In accordance with 45 CFR §46.102(f), approval by an ethics committee and informed consent were not required, because we did not analyze patient-level data. This study follows the Strengthening the Reporting of Observational Studies in Epidemiology (STROBE) reporting guideline.

In June 2018, the FDA limited use of pembrolizumab and atezolizumab to individuals with urothelial cancer who were not eligible for cisplatin-containing therapy. Therefore, we calculated 2 scenarios: 2019 estimations assuming that FDA limits were specific to pembrolizumab and atezolizumab, and 2019 estimations assuming that all immunotherapies approved for urothelial cancers are now limited to patients with high programmed death–ligand 1 expression who are cisplatin ineligible.^4^

Three FDA approvals were granted between August 18 and December 31, 2018 (cemiplimab for cutaneous squamous cell carcinoma and pembrolizumab for hepatocellular and Merkel cell carcinoma). In 2019, atezolizumab was approved for triple-negative breast cancer for individuals who were positive for programmed death–ligand 1 (response rate difference, 20%). For this indication, we assumed that 50% of breast cancer deaths were due to triple-negative breast cancer.^5^ We did not include cemiplimab because death rates from cutaneous squamous cell carcinoma are not included in the American Cancer Society’s Cancer Facts and Figures. Three drugs (atezolizumab, pembrolizumab, and nivolumab) for 4 different cancer types (urothelial, gastric, hepatocellular, and small cell lung cancer) failed to fulfill postmarketing obligations. Because atezolizumab prolongs overall survival in patients with small cell lung cancer, the response for small cell lung cancer tumors was retained in the estimates. Descriptive statistics are provided.

Data were analyzed using Excel statistical software version 2016 (Microsoft Corp). Data analysis was performed from July 2019 to August 2019.

## Results

The estimated eligibility of ICIs in 2019 was 38.5% under the first scenario and 36.1% under the second scenario, translating into an upper bound of 233 790 US patients with cancer. The estimated total responses to these drugs were 11.4% and 10.9% for these respective scenarios. The Figure shows the distribution of estimated eligibility to ICIs, and the Table lists the differences in eligibility estimates between 2018 and 2019. We estimate that up to 9.0% of people who were eligible for ICIs in 2018 were subsequently ineligible because of negative confirmatory trials. The 7.5% reduction in eligibility from our prior 43.6% estimate to the current 36.1% upper-bound estimate is largely because of negative results of phase 3 trials (9.0% reduction), offset by increases in eligibility from other indications, most notably in triple-negative breast cancer (3.5%). Hepatocellular, urothelial, and gastric cancers had the largest negative differences (−4.9%, −2.3%, and −1.8%, respectively).

**Figure.**
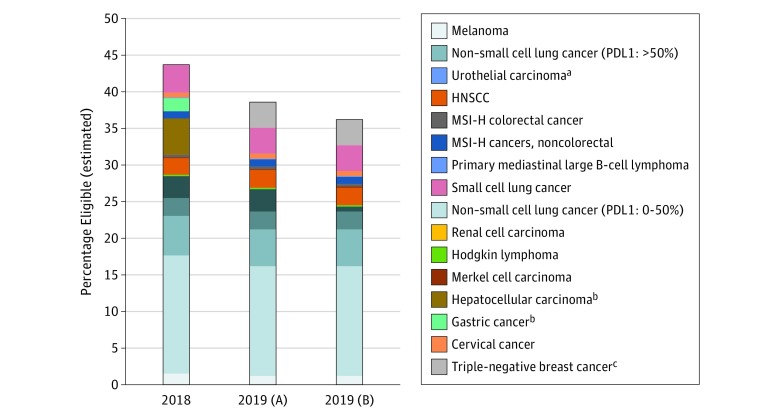
Estimated Eligibility for Immune Checkpoint Inhibitor Drugs in Oncology, 2018 and 2019, Under Several Scenarios The 2019 (A) bar includes limited updates from 2018 to 2019, removing the benefit of hepatocellular and gastric cancers but assuming a benefit to urothelial cancers from immunotherapy drugs (eg, avelumab) that have not had changes to US Food and Drug Administration (FDA) approval status. The 2019 (B) bar includes all updates from 2018 to 2019 and assumes that all immunotherapy drugs for urothelial cancer will have FDA limitations like atezolizumab and pembrolizumab have had. HNSCC indicates head and neck squamous cell carcinoma; MSI-H, microsatellite instability high; PDL1, programmed death–ligand 1. ^a^Cancer type affected by limitations in FDA approval. ^b^Cancer type excluded in 2019 estimates. ^c^Cancer type newly included in 2019 estimates.

**Table.** Estimated Eligibility of Immune Checkpoint Inhibitor Drugs for Years 2018 and 2019

Cancer Type	Estimated Patients Eligible, %			Difference Between 2018 and 2019 Estimates, %^b^
	2018	2019^a^	2019^b^	
Melanoma	1.5	1.2	1.2	−0.3
Non–small cell lung cancer				
Programmed death–ligand 1 level 0%-50%	16.1	15.0	15.0	−1.1
Programmed death–ligand 1 level >50%	5.4	5.0	5.0	−0.4
Renal cell carcinoma	2.5	2.4	2.4	−0.02
Urothelial carcinoma	3.0	3.1	0.7	−2.3
Hodgkin lymphoma	0.2	0.2	0.2	−0.01
Head and neck squamous cell carcinoma	2.2	2.4	2.4	0.2
Merkel cell carcinoma	0.1	0.1	0.1	0
Microsatellite instability high colorectal cancer	0.3	0.3	0.3	0.01
Hepatocellular carcinoma	4.9	0	0	−4.9
Microsatellite instability high cancers, noncolorectal	1.0	1.0	1.0	0
Gastric cancer	1.8	0	0	−1.8
Primary mediastinal large B-cell lymphoma	0.1	0.1	0.1	0
Cervical cancer	0.7	0.7	0.7	0.02
Small cell lung cancer	3.8	3.5	3.5	−0.3
Triple-negative breast cancer	0	3.5	3.5	3.6

^a^ Removing the benefit of hepatocellular and gastric cancers and assuming a benefit to urothelial cancers from immunotherapy drugs (eg, avelumab) that have not had changes to US Food and Drug Administration approval status.

^b^ Removing the benefit of hepatocellular and gastric cancers and assuming that all immunotherapy drugs for urothelial cancer will have US Food and Drug Administration limitations like those that atezolizumab and pembrolizumab have had.

## Discussion

We estimate that up to 9.0% of US patients with cancer may be exposed to ICIs that have had negative phase 3 trial results, which may lower estimated response rates from 12.5% to as low as 10.9%. With faster approval of potentially beneficial drugs, there is also a risk of approving drugs that are later found to be ineffective. Although negative phase 3 trial results are the main reason for lower estimates of eligibility, we also saw lower estimates because of lower numbers of deaths from cancers such as non–small cell lung cancer and melanoma. This was partially offset by increased eligibility to ICIs, most notably for those with triple-negative breast cancer. Our analysis was limited in that estimates were based on clinical trial data and may not be generalizable because of access to medications, off-label use, or response rates in the general population.
